# Synthesis and Evaluation of Engineering Properties of Polymer-Coated Glass Beads

**DOI:** 10.3390/ma16124476

**Published:** 2023-06-20

**Authors:** Boyoung Yoon, Hyunwook Choo, Changho Lee

**Affiliations:** 1 School of Architectural, Civil and Environmental Engineering, Korea University, Seoul 02841, Republic of Korea; by_yoon@korea.ac.kr; 2 Department of Civil and Environmental Engineering, Hanyang University, Seoul 04763, Republic of Korea; choohw@hanyang.ac.kr; 3 Department of Civil Engineering, Chonnam National University, Gwangju 61186, Republic of Korea

**Keywords:** surface modification, polymer, polyurethane-coated glass beads, constrained modulus, maximum shear modulus, rubber-sand mixture

## Abstract

Modern construction projects are often challenging, which has increased the demand for innovative materials that ensure improved safety, durability, and functionality. To explore the potential of enhancing soil material functionality, this study synthesized polyurethane on the surface of glass beads and evaluated their mechanical properties. The synthesis of polymer proceeded according to a predetermined procedure, where the polymerization was confirmed through analysis of chemical structure by Fourier transform infrared spectroscopy (FT-IR) and microstructure observation by a scanning electron microscope (SEM) after complete synthesis. The constrained modulus (*M*) and the maximum shear modulus (*G_max_*) of mixtures with synthesized materials were examined by using an oedometer cell equipped with bender elements under a zero lateral strain condition. Both *M* and *G_max_* decreased with an increase in the contents of polymerized particles due to a decrease in the number of interparticle contacts and contact stiffness induced by the surface modification. The adhesion property of the polymer induced a stress-dependent change in *M* but was observed to have little effect on *G_max_*. Compared to the behavior of the rubber-sand mixtures, polymerized particles show the advantage of a smaller reduction of *M*.

## 1. Introduction

In response to the sophistication of industrial structures and the diversification of social structures, contemporary construction projects require high-rise, large-scale, and lightweight structures, such as skyscrapers and long-span bridges. At the same time, demand for construction materials to improve the lifespan and maintenance efficiency of structures is increasing. As a result, the perception of construction materials is changing to innovative, future-oriented materials that facilitate ongoing maintenance, ensure the safety and durability of buildings, and withstand extreme environmental conditions [1,2,3].

In civil engineering, soil is one of the fundamental and crucial construction materials when starting a construction project. The mechanical properties of soils are a result of extensive interactions between the solid soil particles and the materials filling the void spaces [4,5]. However, because raw soils do not satisfy the required engineering properties for construction projects, many attempts have been made to improve these properties for various applications. These methods can be broadly categorized into physical and chemical stabilization [6]. The former method aims to modify the physical properties or structure of soil, such as through compaction, mixing of anomalous materials, etc. [6,7,8,9]. On the other hand, the latter method improves the soils’ properties by changing the chemical composition of soils by applying cement, lime, etc. [1,2,6,10,11]. However, these conventional methods achieved soil improvement with the help of additional materials such as anomalous materials, cement and lime rather than improving the properties of the geomaterial itself.

Unlike the described methods, this study attempts to improve the fundamental function of geomaterial by synthesizing a polymeric material directly on the surface of glass beads. Among the various polymers used in civil engineering, rubber-like polymers are selected herein because they exhibit unique properties when mixed with geomaterials. Rubber is a ductile material with a strong damping property, and it has been evaluated in various usages such as subgrade material, lightweight backfill, retaining wall, slope stability, and retaining wall when mixed with soil [12,13,14,15]. The inclusion of soft rubber into soils can change the physical properties of soils, including extreme void ratios, hydraulic characteristics, elastic modulus, and friction angle [16,17,18,19,20], as well as the dynamic properties of soils, such as damping ratio and degradation curves of damping ratio and normalized shear [19,21,22,23,24,25]. However, because rubber is highly ductile, an increase in compressibility and decrease in shear modulus are to be expected as the rubber content in a soil-rubber mixture increase. To confirm the polymerization on the surface of the glass beads, FT-IR (Fourier transform infrared spectroscopy) analysis and visual observation of the surface morphology using SEM (scanning electron microscopy) are performed. Then, the effect of polymerized glass bead content on the compressibility and small strain stiffness is investigated through modified one-dimensional compression tests with bender elements. Compared to the physical combination of a rubber-soil mixture, the introduction of surface-modified glass beads with rubber-like polymer is expected to exhibit distinct engineering properties due to modified contact interactions. In particular, it is expected to function as a composite material that exhibits the behavior of both the rubber and the soil. 

## 2. Experimental Program

**Figure 1:**
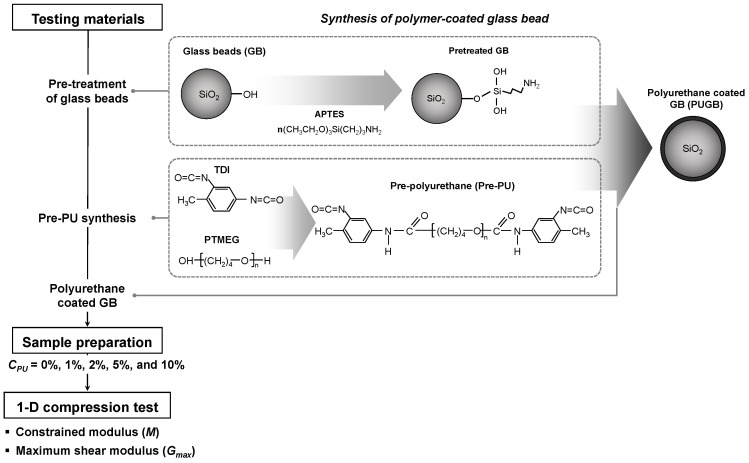
Flow chart of experimental program.

In this study, polyurethane was polymerized at the surface of pretreated mono-sized spherical glass beads. The mechanical properties of the surface-modified particle mixtures were then measured by performing a one-dimensional compression test with an instrumented oedometer cell. The experimental process is summarized in the flowchart outlined in Figure 1, and the details are described in the following sections.

### 2.1. Testing Materials

The mono-sized spherical glass beads (GB, B&K Media Co., Ltd., Hwaseong, Gyeonggi, Korea) were used to avoid particle shape and size effects. The specific gravity (*G_s_*) of the GBs is 2.48 (ASTM D854), and the median particle size (*D_50_*) of GB is 0.51 mm (ASTM D6913). Other index properties are listed in Table 1. Before the synthesizing process, the pretreatment of GB was conducted using the silane coupling agent: 3-aminopropyltriethoxysilane (APTES, Sigma Aldrich, St. Louis, MO, USA). For the pre-polyurethane synthesis, toluene diisocyanate (TDI; the isomer mixture ratio between 2,4- and 2,6-TDI was 80:20) (Sigma Aldrich) and poly tetramethylene ether glycol (PTMEG, 1000 g/mol) (Sigma Aldrich) were prepared. Two types of TDI were mixed in the weight ratio of 80:20. APTES and PTMEG were dehydrated under vacuum drying at 80 °C for 12 h before use [26].

### 2.2. Synthesis of Polyurethane-Coated Glass Bead (PUGB)

Surface modification of the GBs was achieved through a rapid reaction between the NCO group and OH group, which allowed the silanol group (Si-OH) to react with the terminal isocyanate groups (R-N=C=O) of the polyurethane prepolymer (pre-PU) (Figure 1) [26]. Before synthesis, the GBs were first immersed in 1% (*w*/*w*) APTES in anhydrous toluene (99.8%, Samchun Chemicals, Seoul, Korea) [27] and stirred for 1 week to enhance the number of silanol groups on their surfaces. Thereafter, the GBs were washed with anhydrous toluene, deionized water, and ethanol (Samchun Chemicals). The pre-PU was prepared using the method described by Chen et al. [26]: 0.052 mol of the dehydrated PTMEG was added to a three-necked flask and stirred at 80 °C for 30 min. Then, 0.103 mol of TDI was added to the flask to react with the PTMEG. The mixture was stirred for more than 1 h to induce an adequate reaction. The surface-modified GBs were then prepared by stirring a mixture of 1% (*w*/*w*) of pre-PU with the silane-treated GB at 80° for 24 h.

### 2.3. Sample Preparation and Experimental Procedure

The polyurethane-coated GB (PUGB) content in the mixture (*C_PU_*) was defined as the ratio of the weight of PUGB (*W_PUGB_*) to the weight of the total mixture (*W_t_*), as follows:

(1) $$C_{PU}=\frac{W_{PUGB}}{W_{t}}=\frac{W_{PUGB}}{W_{GB}+W_{PUGB}}$$

where *W_GB_* is weight of the pure GBs. Five different mixtures were prepared with different *C_PU_* values of 0% (i.e., pure GB), 1%, 2%, 5%, and 10% to explore the effects of *C_PU_* on the behavior of the mixture. An instrumented oedometer cell mounted with the bender elements was used to investigate the stress-dependent and small-strain characteristics of the mixtures under the zero lateral strain condition. The inner diameter and height of the cell were 100 mm and 72.5 mm, respectively. A pair of bender elements was installed on the top cap and another pair on the bottom plate to measure the shear wave velocity (*V_s_*). The shear wave velocity was calculated based on the first arrival time (*t_first_*) and tip-to-tip distance between the bender elements (*L_tip-to-tip_*) [28]. A predetermined amount of the mixture was poured into the cell. For each *C_PU_*, the mixtures were consistently prepared to three different relative densities (*Dr*) of 40%, 60%, and 80%. The mixture was loaded to 450 kPa with a load increment ratio of 1 and unloaded to ~1 kPa. A linear variable displacement transducer (LVDT) with a precision of 0.001 mm was used to monitor the vertical displacement of the mixtures during the loading and unloading steps. Vertical settlement and shear wave were measured in each step.

## 3. Experimental Results

### 3.1. Analysis of Chemical Structure

**Figure 2:**
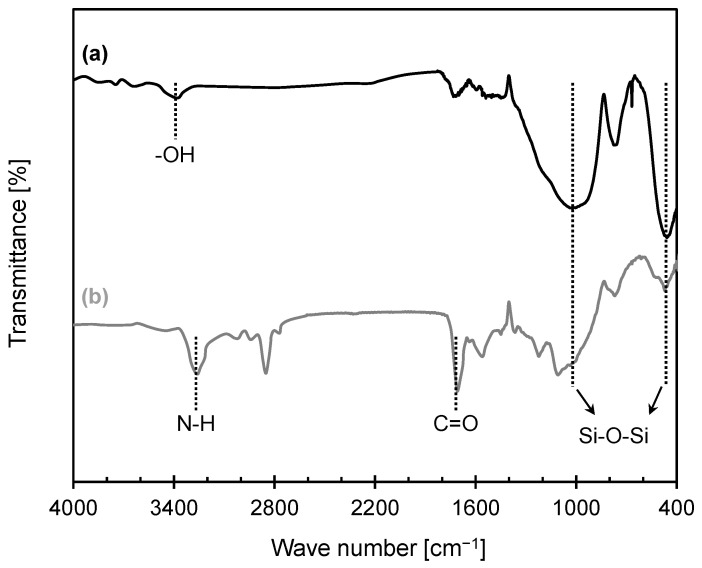
FT-IR spectrum of (a) pretreated GB and (b) GB after synthesize process (i.e., PUGB).

Figure 2 presents a comparison of the FT-IR spectra of the GBs before and after the synthesis process. Since the FT-IR analysis is based on the interaction of infrared radiation with molecules, the response of the sample at different wavenumbers are the characteristics of different functional groups present in the sample. Therefore, the analysis of FT-IR enables the identification of compounds. In particular, the -OH functional group of pretreated GB and N-H and C=O groups of PUGB are of interest, as they exhibit the characteristics of chemical bonds described in Figure 1. The spectrum of pretreated GB exhibited a clear peak close to wavenumbers 1100 cm^−1^ and 470 cm^−1^, assigned to the silica network (Si-O-Si) [29]. Moreover, the peak at around 3450 cm^−1^, representing the OH groups on the GB surface [30], confirmed that APTES effectively enhanced the silanol groups, which results in a higher chance of reacting with the pre-PU. Notably, the PUGB particles exhibited adsorption peaks at 3330 cm^−1^ and 1730 cm^−1^, which originated from the stretching vibrations of the N-H and C=O groups, thereby confirming the successful pre-PU coating on the pretreated GB surface (Figure 1) [26]. The observed attenuated peak at 3450 cm^−1^ for PUGB also supported the occurrence of a chemical reaction between the OH groups on the GB surface.

### 3.2. Microstructure Observation

**Figure 3:**
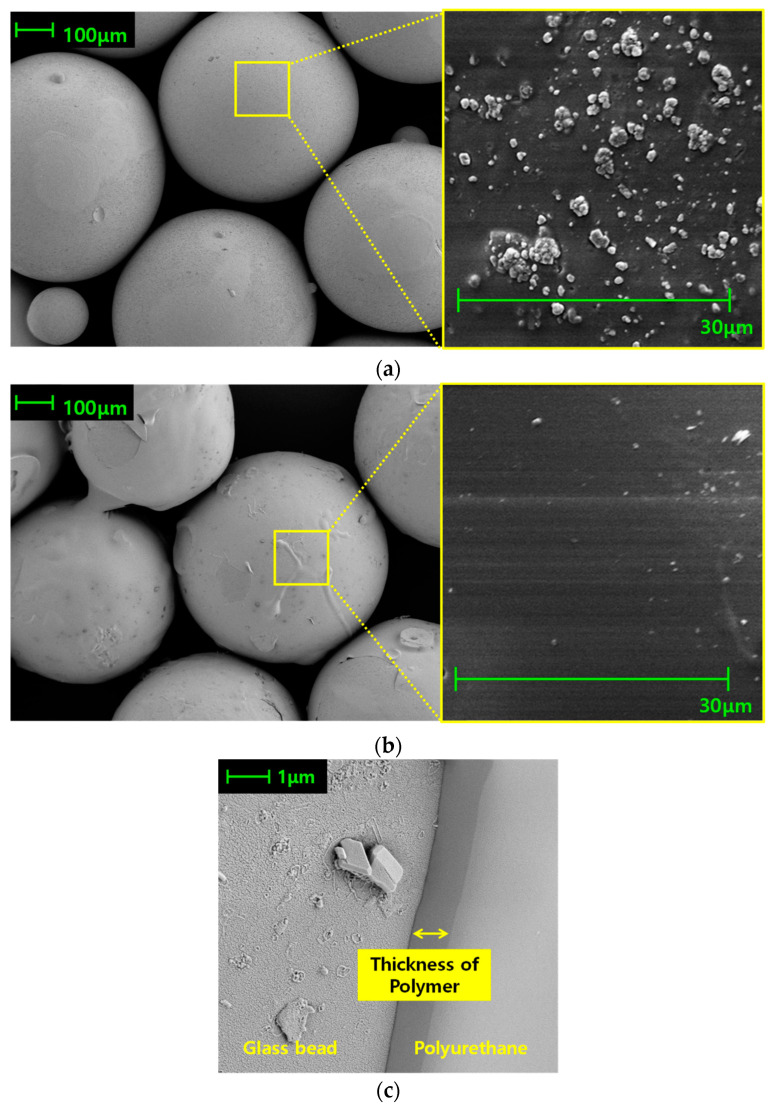
SEM images of (**a**) pure GB and (**b**) PUGB with closeups in images to evaluate the relative surface roughness and (**c**) cross-section of PUGB showing the thickness of polymer layer.

Since the polymer synthesis was confirmed by FT-IR analysis, the morphology changes of the GB surface before and after polymer synthesis were observed by SEM images (Figure 3). Figures on the 100 μm scale indicate that surface modification by the polymerization was unaffected by the shape or size of the pure GB particles and that the synthesized polymer completely covers the GBs. The close-up image of the pure GB surface (right side of Figure 3a) reveals the presence of wedge-shaped protuberances on the surface, whereas in the case of PUGB (right side of Figure 3b), the synthesized polymer covers them, resulting in a relatively smooth surface. The thickness of coated polymer on the GB was measured to be nearly constant at ~0.9 μm (Figure 3c). Accordingly, it was inferred that the surface modification did not alter the size distribution of the GBs, but the aforementioned changes in surface roughness may lead to different behaviors of the PUGB-GB mixtures depending on their *C_PU_*.

### 3.3. Variation of Extreme Void Ratios

**Figure 4:**
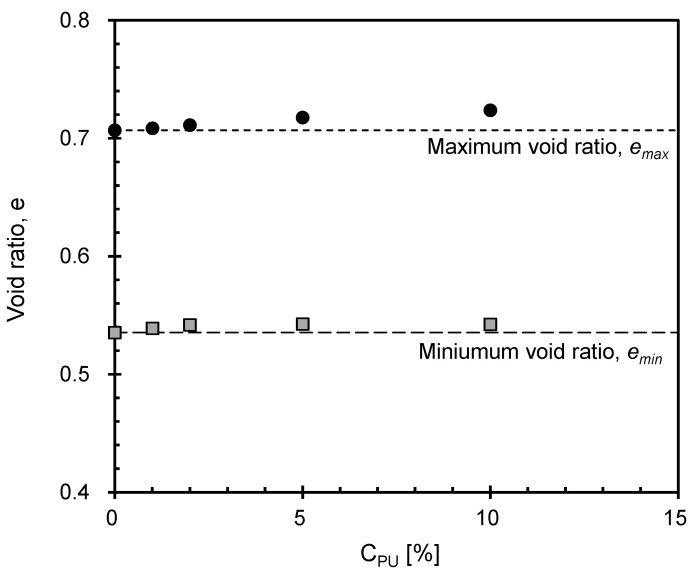
Variation of extreme void ratios with *C_PU_*. Each dashed lines indicate *e_max_* and *e_min_* at *C_PU_*, respectively = 0% (i.e., pure GB).

The variations in maximum void ratio (*e_max_*) and minimum void ratio (*e_min_*) of the PUGB-GB mixtures having various *C_PU_* values are illustrated in Figure 4. The change in *e_min_* with *C_PU_* was relatively insignificant, while *e_max_* increased slightly as *C_PU_* increased. This may be attributed to the increased contact area between the particles resulting from reduced surface roughness due to surface modification, which enhanced the influence of the intrinsic adhesive properties of the polymer [31,32]. Particularly, Yu et al. [33] demonstrated that the adhesive forces between flat glass and polyurethane increased as the particle contact number increased. Thus, an increase in *C_PU_* leads to an increase in particle adhesion, resulting in higher *e_max_*. However, because the adhesion forces were not high enough to withstand the stress exerted during vibratory table testing (i.e., *e_min_* measurement), an insignificant effect of *C_PU_* was observed.

### 3.4. Vertical Compressibility at Zero-Lateral Strain

**Figure 5:**
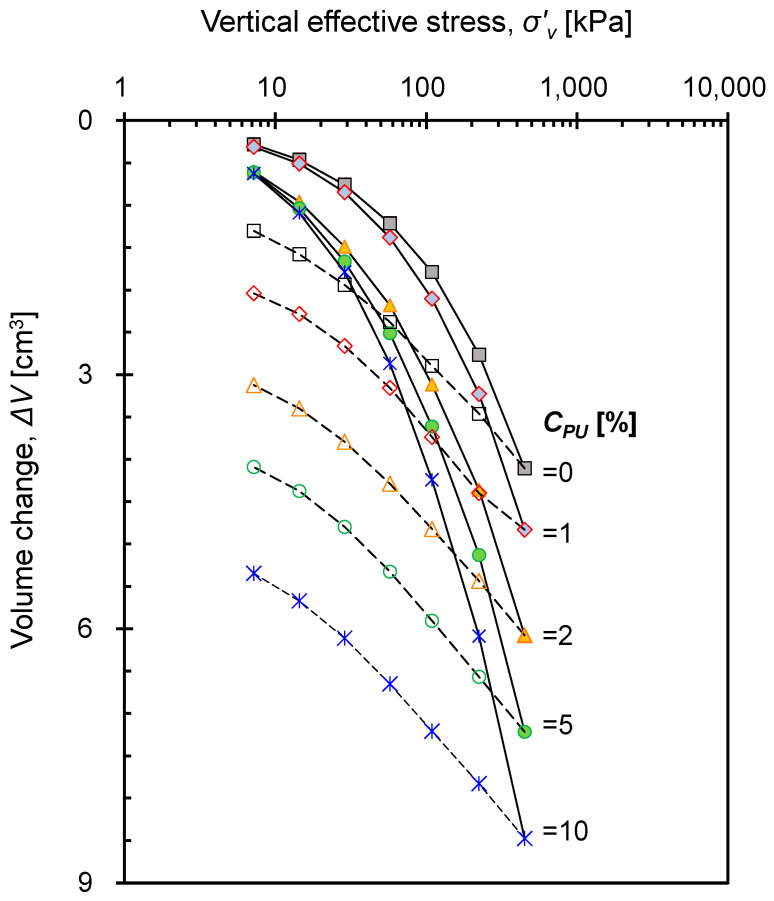
Volume change for the mixtures of *Dr* = 40% during loading and unloading stages at a zero-lateral strain condition.

Figure 5 shows the evolution of the volume change (*ΔV*) behavior of the PUGB-GB mixtures determined on the basis of settlement during loading and unloading under the zero-lateral strain condition. At a given relative density, *ΔV* of the mixture increased as the applied vertical stress and *C_PU_* increased. The volumetric change refers to the particle rearrangement behavior induced by particle rolling and sliding [34]. Accordingly, the increase in *ΔV* with *C_PU_* reflects that the PUGB particles were rearranged more easily than the GB particles, which was attributed to the increased initial void ratio and reduced surface roughness. The aforementioned adhesion forces of polymer may initially resist the particle rearrangement; however, it rather served to aggregate the particles around the PUGB with vertical stress increment, leading to higher *ΔV* with increasing *C_PU_*.

**Figure 6:**
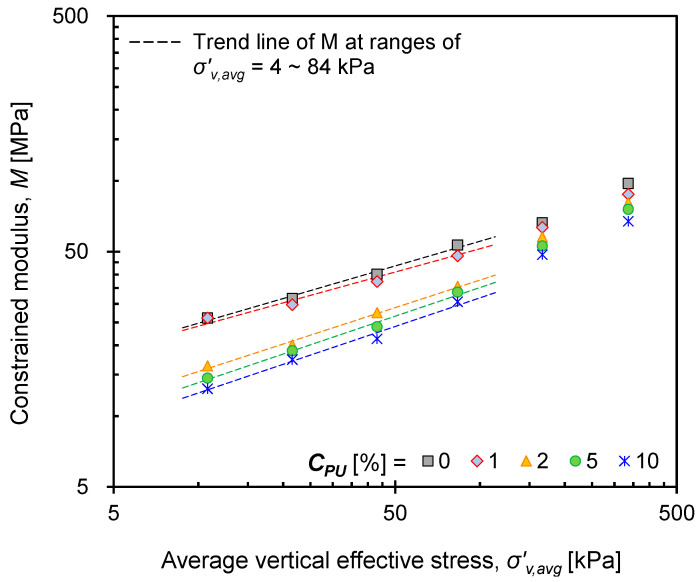
Evolution of the constrained modulus with the relative density during loading (shown only in the case of *Dr* = 60%).

The constrained modulus (*M*) was calculated as the slope of the vertical effective stress–strain curve, where the measured value represents *M* at the average vertical stress (*σ’_v,avg_* )of two vertical stresses applied sequentially. Figure 6 only shows the variation of *M* with the average vertical stress at *Dr* = 60%, as consistent variation trends were observed at varying *Dr*. As expected, *M* decreased with *C_PU_* because of the increased number of particles with reduced surface roughness. However, the *C_PU_* = 1% mixture exhibited a similar variation in *M* as that of the *C_PU_* = 0% mixture (i.e., pure GB), reflecting the insignificant effect of PUGB particles on mixture behavior.

The stress-dependent variation of *M* at different *C_PU_* values were observed through the trend line of *M* at ranges of *σ’_v,avg_* = 4~84 kPa. For mixtures with low *C_PU_* (≤1%), the *M* at *σ’_v,avg_* > 100 kPa follows similar trends to those of trend lines. However, for mixtures with *C_PU_* > 1%, the *M* at *σ’_v,avg_* > 100 kPa deviates from the trend line and increases rapidly, approaching *M* of pure GB. It is inferred that the adhesion of the polymer brings the pure GBs into closer contact with the PUGBs, which initially increases the mixture compressibility. However, when a certain stress level at which no further interactions between PUGBs are induced, the fabric of GBs may start to govern the mixture behavior. As a result, the mixtures exhibit GB-like behavior and approach the *M* value of pure GB. Although further study is needed to determine the precise onset of interactions, the stress-dependent behavior of PUGB-GB mixtures with *C_PU_* > 1% was observed at *σ’_v,avg_* ~ 100 kPa in this study.

### 3.5. Shear Wave Velocity V_s_

**Figure 7:**
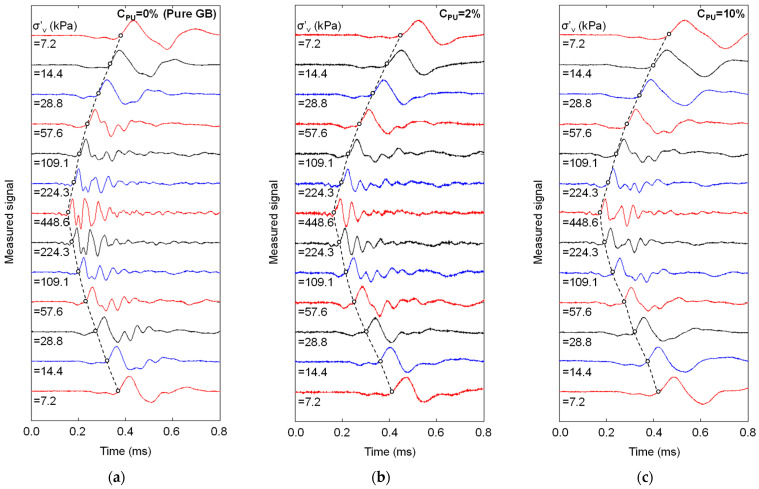
Time series of the shear wave traces for selected mixtures with *Dr* = 60% during loading and unloading: (**a**) *C_PU_* = 0%; (**b**) *C_PU_* = 2%; and (**c**) *C_PU_* = 10%.

The propagation of shear wave traces during the loading and unloading stages of the selected mixtures with the initial *Dr* of 60% are plotted in Figure 7. The first arrival time decreased, and the resonant frequency increased with vertical stresses [28,35,36]. As the *C_PU_* increased, the first arrival time of the mixture increased under the same vertical effective stress, indicating that the polymer coating on the GB surface lowered the contact stiffness. Nonetheless, the resonant frequency of the signal decreased in a negligible range with the increasing *C_PU_*, indicating insignificant changes in the global material properties with *C_PU_*.

**Figure 8:**
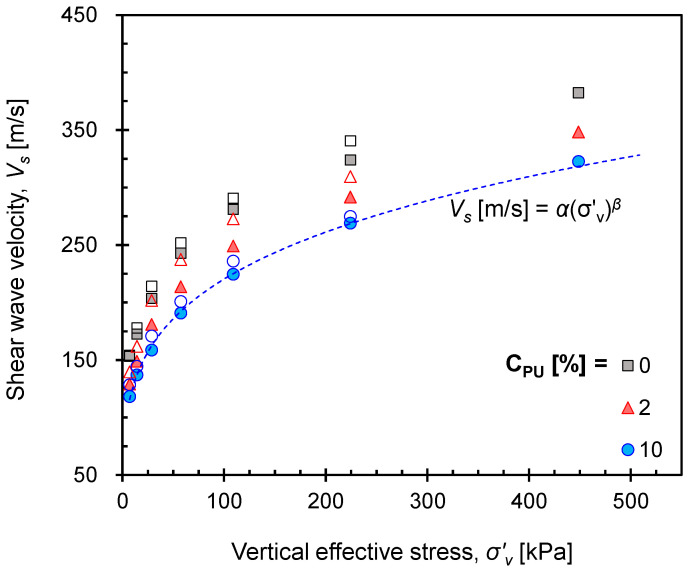
Evolution of measured shear wave velocity for selected specimens with *Dr* = 60% during loading and unloading. Solid and hollow markers indicate loading and unloading stages, respectively. Note that the dotted line in the figure denotes shear wave velocity for *C_PU_* = 10% in loading stage calculated by Equation (2).

The shear wave velocity was then calculated from the measured signals and travel distances. As the increases in the applied stress and relative density induced higher contact area followed by higher contact stiffness, a higher shear wave velocity was observed. On the contrary, because the increase in *C_PU_* decreased the contact stiffness between particles, a lower shear wave velocity was measured with *C_PU_*, regardless of the initial *Dr* and loading/unloading states (Figure 8).

## 4. Discussion

### 4.1. Relationship between α and β

**Figure 9:**
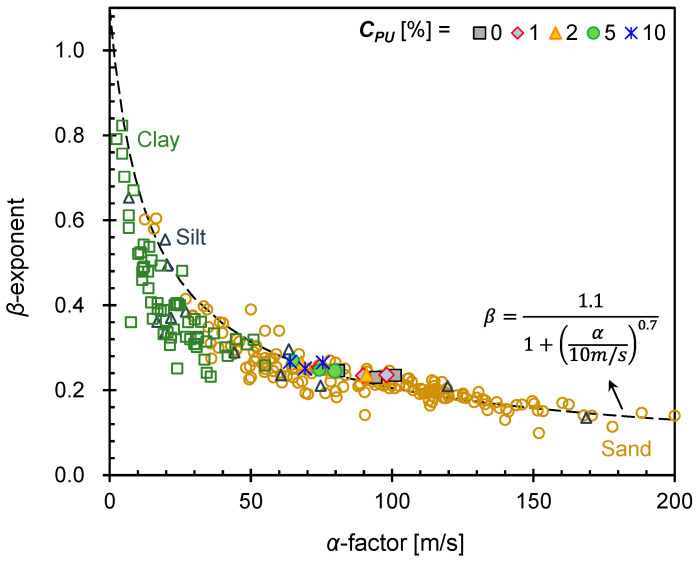
Relationship between α-factor and β-exponent. Hollow markers in the figure indicate relation between the α-factor and the β-exponent for different soil types from previous studies [42,43,45,46,47]. Hollow squares, triangles, and circles denote clay, silt, sand, respectively.

The shear wave velocity can be expressed as a power function of the effective stress [37,38,39,40], as follows:

(2) $$V_{s}=\alpha {\left(\frac{\sigma_{v}^{’}}{1\;kPa}\right)}^{\beta }$$

where the *α*-factor [m/s] is the shear wave velocity at 1 kPa confinement, and the *β*-exponent indicates the stress sensitivity of the shear wave velocity. Both *α* and *β* were determined experimentally on the basis of particle packing, fabric, mineral properties, and contact characteristics, such as the coordination number and contact stiffness [41]. Therefore, *α* and *β* have a range of values that depends on both intrinsic properties and state conditions of the given soil types: clay, silt, and sand [42,43,44]. Comparing the relationship between the α-factor and *β*-exponent obtained in this study with data of different various geomaterials [42,43,45,46,47] can be helpful in identifying the behavior of PUGB-GB mixtures (Figure 9). The most recent comprehensive work regarding the *α*-*β* relationship is suggested by Ramirez et al. [42], where the suggested relationship is plotted together in Figure 9. For denser and coarser materials, a higher *α*-factor and a lower *β*-exponent are expected due to the smaller changes in the interparticle contact number with increasing stress [41]. The values of the *α*-factor and *β*-exponent of the tested pure GB indicate that the behavior of pure GB is equivalent to that of pure sand. Furthermore, the increase in *C_PU_* did not modify the global properties of the materials since the relation of *α* and *β* parameters still lies on the suggested trend line. Also, it is notable that PUGB-GB mixtures exhibit sand-like parameters, regardless of *C_PU_*_,_ confirming the findings in Figure 7. Still, the increase in *C_PU_* decreased the contact stiffness, resulting in a decrease and an increase in the *α*-factor and *β*-exponent, respectively. The relationships of *α* and *β* obtained in this study are adequately consistent with the suggested trend line for soils, despite the change in *C_PU_*. This finding reinforces that the PUGB can still act as a geomaterial.

### 4.2. Variation and Transition Behavior of M and G_max_ with C_PU_

**Figure 10:**
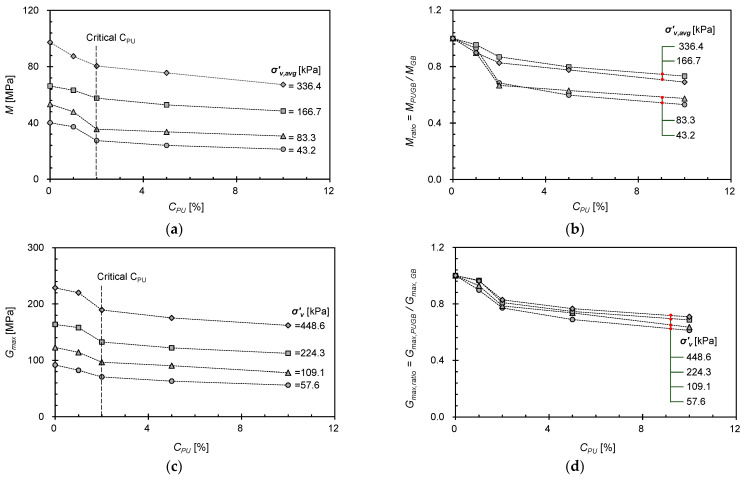
Effect of *C_PU_* on modulus ((**a**) *M* and (**c**) *G_max_*) and modulus ratio ((**b**) *M_ratio_* and (**d**) *G_max,ratio_*) of PUGB mixtures at various stress level. Only the PUGB mixtures with *Dr* = 60% are shown.

As examined, the behaviors of the PUGB mixtures were controlled by *C_PU_*: the constrained modulus (*M*) represents the stress-dependent deformation due to fabric and skeletal changes under intermediate strain, while the maximum shear modulus (*G_max_*) was influenced by the interparticle contact characteristics under small strains [36,48]. The effects of *C_PU_* on each modulus at various stress levels are compared in Figure 10a,c. As the total particle contact number increased with the (average) vertical effective stress (*σ’_v,_* or *σ’_v,avg_*), followed by an increase in interparticle contact stiffness, both *M* and *G_max_* increased. As *C_PU_* increased, *M* and *G_max_* decreased because the surface roughness decreased, which facilitated particle rearrangement and reduced particle contact stiffness. The variation of *M*, however, showed stress-dependent behavior that could be distinguished at *σ’_v,avg_* = ~100 kPa, as summarized in Figure 6. To evaluate the stress-dependent behaviors of both *M* and *G_max_*, the modulus ratio (*M_ratio_* = *M_PUGB-GB_*/*M_GB_* or *G_max,ratio_* = *G_max,PUGB-GB_/G_max,GB_*), which is the ratio between the moduli of the mixture and those of pure GB, was compared (Figure 10b,d). The comparison results clearly show that the variation of the *M_ratio_* for *C_PU_* is divided at *σ’_v,avg_* = ~100 kPa (Figure 10b), while the constant trend of the *G_max,ratio_* is according to the stress level (Figure 10d). Thus, it can be interpreted that the effect of the polymer coating on the GB surface depends on both stress and strain. Figure 10 clearly shows a significant decrease in *M* and *G_max_* at *C_PU_* = ~2%, after which the effect of *C_PU_* was negligible (Figure 10a,c). Therefore, *C_PU_* = ~2% can be assumed to be the critical *C_PU_* for PUGB particles to start controlling the mixture behavior.

**Figure 11:**
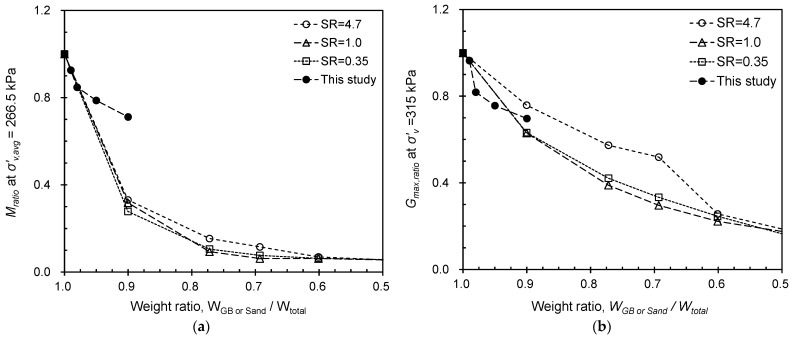
Comparison of modulus ratio: (**a**) *M_ratio_* and (**b**) *G_max, ratio_* of PUGB mixtures with rubber-sand mixtures performed by Lee et al. [36] represented by hollow markers: circles, triangles and squares denote rubber-sand mixtures with *SR* = 4.7, 1.0 and 0.35, respectively.

The modulus ratio such as *M_ratio_* and *G_max,ratio_* of tested PUGB-GB mixtures were then compared with those of rubber-sand mixtures reported by Lee et al. [36]. Lee et al. [36] performed the one-dimensional compression test of rubber-sand mixtures prepared with various size ratios (*SR* = medium diameter ratio between rubber and sand = *D_50,rubber_*/*D_50,sand_*) and volumetric sand fractions (=*V_sand_/V_total_*). The modulus ratios (*M_ratio_* and *G_max,ratio_*) of tested PUGB-GB mixtures were plotted with the modulus ratios of the rubber-sand mixtures at three representative *SRs* including larger (*SR* = 4.7), similar (*SR* = 1.0), and smaller (*SR* = 0.35) rubber compared to sand and are shown in Figure 11. Note that the *SR* of PUGB is close to 1.0. The weight fractions of the rubber-sand mixtures are calculated based on the volumetric sand fraction given the specific gravity of sand (*G_s,sand_* = 2.62) and rubber (*G_s,rubber_* = 1.16). The modulus ratio at a specific stress level is computed by averaging the modulus ratio of applied stresses that come before and after the given stress, in sequential order. Figure 11 demonstrates that PUGB-GB mixtures possess a significant advantage in terms of their larger *M_ratio_*, indicating reduced compressibility compared with rubber-sand mixtures. Furthermore, the *G_max,ratio_* of PUGB-GB mixtures is similar to that of rubber-sand mixtures. These results suggest that PUGB-GB mixtures provide improved performance in terms of compressibility while maintaining *G_max_* similar to that of pure geomaterials.

**Figure 12:**
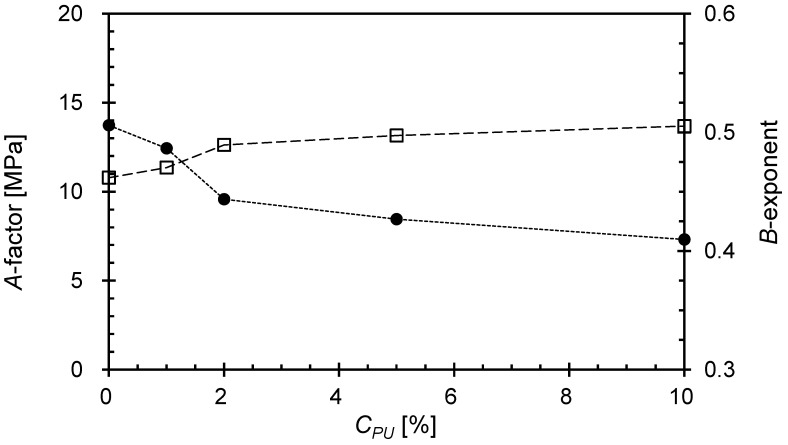
Variation of *A*-factor (solid marker) and *B*-exponent (hollow marker) of Equation (3) according to *C_PU_*.

Further analysis on *G_max_* was performed to reveal a “measure of state” because *G_max_* is known to be controlled by the nature of interparticle contact and coordination [41]. The *G_max_* values of uncemented granular materials can be predicted using the following semiempirical power function in conjunction with the effective vertical stress and mass density of the material (ρ), as suggested by [28]:

(3) $$G_{max}=\rho V_{s}^{2}=A{\left(\frac{\sigma_{v}^{’}}{1\;kPa}\right)}^{B}$$

where *A* and *B* = experimentally determined factor and exponent, respectively. The *A*-factor denotes *G_max_* of the mixture at the $\sigma_{v}^{’}$ of 1 kPa, which is related to the initial packing of the mixtures, while the *B*-exponent indicates the stress sensitivity of *G_max_* reflecting the contact behavior and fabric changes within mixtures [41]. Generally, the *A*-factor increases while the *B*-exponent decrease as the stiffness of the mixture increases [41,49]. Therefore, the increase and decrease of the *A*-factor and *B*-exponent with the *C_PU_* increment indicates the decrease in material stiffness with *C_PU_* (Figure 12). Moreover, the remarkable changes in the *A*-factor and *B*-exponent are observed at *C_PU_* = ~2%, which is the critical *C_PU_*. Still, the *B*-exponent appeared to be similar to that of pure GB, thus confirming that the introduction of PUGB particles did not alter the global behavioral properties of the mixtures within the tested *C_PU_* range.

### 4.3. Potential Use of Surface-Modified Materials as Geotechnical Materials

The behaviors of the PUGB-GB mixtures could lead to several advantages over those of the conventional soil-rubber mixtures. An increase in the rubber content of a soil-rubber mixture increases the damping ratio of the mixture owing to the material properties of rubber [50,51,52], but at the same time increases the compressibility of the mixture [36,53]. However, for PUGB-GB mixtures, the increase in compressibility was not as large as those for soil-rubber mixtures despite the increase in *C_PU_* (Figure 11). Although it was necessary to confirm the dynamic behavior according to the shear strain rate through additional resonant column tests, it is presumed that the dynamic behavior of the PUGB-GB mixture will improve owing to the influence of PU polymer. Therefore, the PUGB-GB mixtures can possibly be utilized as new geomaterials that overcome the shortcomings of traditional soil-rubber mixtures.

## 5. Conclusions

This study aims to improve the functionality of glass beads (GB) by synthesizing polyurethane on their surface while also evaluating the mechanical properties of their mixtures with polymerized particles (PUGB). One-dimensional compression tests using an instrumented oedometer cell equipped with bender elements were performed to investigate the effect of the contents of the polymerized particles (*C_PU_*) on the constrained and shear moduli of their mixtures. The key findings can be summarized as follows:

(1) Polymer coating on the GB surface reduced the surface roughness, increasing the contact area of the particles as the *C_PU_* increased. The intrinsic adhesion of the polymer increased the maximum void ratio of PUGB-GB mixtures. However, the minimum void ratios of the mixtures showed negligible changes with *C_PU_* because the adhesion could not withstand the stress applied during the vibration table test.
(2) Lower constrained modulus (*M*) was observed for the PUGB-GB mixture with higher *C_PU_* as the vertical deformation increased due to an increase in the initial void ratio of the mixture and a decrease in surface roughness.
(3) The shear wave velocity (*V_s_*) of the mixtures decreased with *C_PU_* because coated polymer reduced the contact stiffness between particles.
(4) The critical *C_PU_*, at which the PUGB particles start to become involved in the load-carrying skeleton of the mixture, was found to be ~2%. This finding was also confirmed by the *A*-factor and *B*-exponent values, which describe the measured state of the maximum shear modulus (*G_max_*), which are also found to vary significantly at *C_PU_* = ~2%.
(5) The stress- and strain- dependent behavior of the PUGB-GB mixture is demonstrated by comparing the evolution of modulus, where the trends of *M* exhibit changes at certain average stress, while variation trends of *G_max_* remain constant over the ranges of applied stress.
(6) Comparing the behavior of PUGB-GB mixtures and the rubber-sand mixtures, it can be seen that the introduction of PUGB particles leads to a smaller *M* reduction with a similar *G_max_* of the mixture compared to rubber.

This study evaluated the potential application of polymer coating for enhancing the performance of PUGB-GB mixtures. Further studies on the analysis of the dynamic behavior of PUGB-GB mixtures are expected to expand their promising prospects for various applications.

## Figures and Tables

**Table 1:** Index properties of used materials in this study.

Material		Glass Bead (GB)	Device/Technique
Specific gravity, *G_S_*		2.48	Pycnometer (ASTM D845)
Median particle size *D_50_* [mm]		0.51	Sieve (ASTM D6913)
Coefficient of uniformity, *C_u_*		1.22	-
Coefficient of curvature, *C_c_*		0.97	-
Extreme void ratio	Minimum void ratio, *e_min_*	0.55	Vibratory Table (ASTM D4253)
	Maximum void ratio, *e_max_*	0.69	Funnel (ASTM D4254)

## Data Availability

All data, models, and code generated or used during the study appear in the published article.
